# An overview of the treatment of cutaneous leishmaniasis

**DOI:** 10.12703/r/9-28

**Published:** 2020-12-22

**Authors:** Talissa F Garza-Tovar, Marco I Sacriste-Hernández, Eder R Juárez-Durán, Roberto Arenas

**Affiliations:** 1 Dermatology Department, Hospital of Specialties 25 IMSS, Monterrey, Nuevo León; 2 Mycology Section, "Dr. Manuel Gea Gonzalez" General Hospital, Mexico City, Mexico

**Keywords:** Leishmaniasis, Treatment, Antimonials

## Abstract

Leishmaniasis is a neglected tropical disease caused by species of *Leishmania,* with a broad spectrum of clinical manifestations, such as cutaneous, visceral, and mucocutaneous presentations. Many drugs are used for its treatment, and a current effective one is a pentavalent antimonial, especially in developing countries. In this review, we discuss recent proposed therapies as well as their side effects.

## Introduction

Leishmaniasis is a neglected tropical disease caused by species of *Leishmania* and transmitted to humans by the bite of a sandfly, mainly *Phlebotomus* and *Lutzomyia* (Old World and New World, respectively)^1^. It is a major public health problem with a broad spectrum of clinical manifestations and epidemiological diversity with different degrees of severity that depend on the involved intracellular parasite** and the immune response of the host. It has a wide distribution and is endemic in circumscribed areas in Northeastern Africa, Southern Europe, the Middle East, Mexico, and Central and South America^1^. In its treatment, many drugs are used, but around 50% of satisfactory clinical and microbiological results for almost all clinical forms are achieved with a current pentavalent antimonial. This review evaluates recently proposed treatments and their side effects.

## Local and intralesional therapy

Local therapies have been recommended by the World Health Organization (WHO), the Pan American Health Organization (PAHO), and other experts as an alternative to systemic drugs in patients with at least four lesions smaller than 4 cm in diameter, especially when the face or joints are not affected. Usually, they are easy to use and have a lower risk, toxicity, and cost compared to traditional therapies. Local treatment includes thermotherapy, cryotherapy, and topical and intralesional drugs. The last approach is effective and safe in localized forms^2–4^.

### Intralesional antimonials

The use of intralesional therapy with antimonials was introduced with the aim of reducing adverse effects compared to their systemic administration. It was recommended as an acceptable therapeutic alternative for New World leishmaniasis by the WHO Expert Committee on Leishmaniasis in 2010 and by the PAHO Expert Committee on Leishmaniasis in 2013^5,6^. In a systematic review of the efficacy of pentavalent antimoniate intralesional infiltration for cutaneous leishmaniasis (CL), the authors found an efficacy rate in the Old World of 75% with intralesional pentavalent antimony (SbV) (higher when it was combined with cryotherapy), 83% with sodium stibogluconate (SSG), and 68% with meglumine antimoniate (MA). In the New World, the efficacy rate was 77% with intralesional infiltration SbV, 61% with SSG, and 82% with MA^7^. We must consider that this therapy requires the infiltration of each lesion so is not for all cases of CL. The recommended dose is 1–5 mL (applied in five sites per lesion) every 3–7 days until healing; the only adverse events reported were local irritation, pain, edema, erythema, and pruritus^3,8^.

### Thermotherapy/heat therapy

Thermotherapy is a local treatment based on studies that have demonstrated *in vitro* growth inhibition of *Leishmania* in macrophages at temperatures >39°C; moreover, heat and the immediate collagen contraction stimulates the destruction of parasites^2^.** Thermotherapy can be administered with infrared light, laser, or direct electrical stimulation. Radiofrequency has advantages over other local treatments for Old and New World Leishmaniasis^8,9^. One is that radiofrequency waves penetrate uniformly to a depth of 4 mm, so the amastigote can be heated with high temperatures without damaging the surrounding skin, and waves can be applied locally with portable, battery-operated, and localized current fields (ThermoMed). This device is safe compared with other traditional treatments and easy to use in rural areas without electricity; however, the thermotherapy machine is expensive. Heat therapy is recommended for lesions caused by any species of *Leishmania*, and the main side effects are pain, itching, burning sensation, and even second-degree burns^2,9,10^. It can be used with systemic treatments and is an alternative approach in pregnant women with localized leishmaniasis or patients who have contraindications to systemic therapy^3^. It has been reported that a single application of radiofrequency thermotherapy at 50°C for 30 seconds is an effective and safe treatment, with cure rates of 87 to 98%. The discrepancy between the cure rates may be due to other factors like host immunological response, nutritional status, and different *Leishmania* spp.^9,11,12^*.* It is important to consider that this therapy generates a local thermal dose, so it might not be possible to cure distant lesions, such as in patients with lymph node and mucosal involvement , especially in regions with a high prevalence of mucosal leishmaniasis^9,13^.

### Cryotherapy

Cryotherapy is a physical therapy that uses subzero temperatures and is effective because all Leishmania species are thermosensitive. Liquid nitrogen at –196°C is applied directly to the lesion, reaching a lethal temperature^14^. It has to be applied to the skin for 15–20 seconds, extending 1–2 mm outside of the lesion, and has to be repeated three times per session every 3 weeks. The result of treatment in local tissues is direct damage, with the destruction of amastigotes, which induces an immune response to the liberation of cellular antigenic substances^3,15–17^. Tolerance to the application of cryotherapy is adequate, and it is a low-cost treatment with few side effects, such as erythema, edema, and residual hypopigmentation or hyperpigmentation^2,18^. However, despite the advantages of this local therapy, there are large discrepancies among the findings of some controlled trials: some report success in more than 95% of cases, while in some others the percentage of successful cases is 27%^16^. A systematic review of the efficacy of pentavalent antimoniate intralesional infiltration therapy for CL found that the cure rate with antimony intralesional infiltration combined with cryotherapy was higher than antimony intralesional infiltration alone (odds ratio [OR] 3.14, *P* = 0.013)^7^. The above is correlated with several studies that have shown that CL responds better to the combination of cryotherapy with antimony intralesional infiltration than each technique alone^8,15,19^.

### Lasers

***CO_2_ laser*.** CO_2_ laser therapy for CL causes a specific thermolysis effect on infected tissue and has fewer side effects on normal skin^2,8,20^. In Iran, researchers compared the efficacy of CO_2_ laser with a combination of cryotherapy and intralesional MA; they showed that the first approach was more effective, with a complete response in up to 93% of patients. They used a CO_2_ laser system with a maximum power of 30 W, pulse duration of 0.01–1 second, and a mode of continuous wave^20^. In Israel^21^, 10 pediatric patients with a diagnosis of CL were treated with fractional CO_2_ laser (spot size 120 mm, fluence 15–100 mJ, and density 3–5%) followed by immediate topical application of SSG; 90% achieved clinical cure with good final cosmetics. The CO_2_ laser is safe and effective with few sessions and is associated with mild side effects, such as hyperpigmentation and hypertrophic scars^2,21^.

***Argon laser*.** A review of the literature on the application of laser for the treatment of CL found one report of argon laser as treatment^22^. Rakaheev *et al*. reported complete clinical cure of a patient who had failed antibiotic treatment; they treated the patient with six sessions of argon laser therapy with intervals of 4–5 days^23^. As this is the only study reporting on this treatment modality, more studies are needed.

***Erbium glass laser*.** Mashayekhi *et al*. reported the use of erbium glass laser for the treatment of CL^24^. The study was conducted in Iran, and the authors treated 14 patients with 20 lesions of Old World CL (OWCL) using erbium glass laser 1540 nm weekly (10 mm spot size handpiece with 10 ms pulse duration and 50–70 mJ/cm^2^ fluence in four passes). Only nine patients with 12 lesions ended the protocol, and 91.66% of the lesions improved completely at 6 weeks of treatment. The side effects reported were pain during the procedure, erythema, and edema. The authors concluded that it may be a promising method for the treatment of CL^24^.

***Neodymium-doped yttrium aluminum garnet laser*.** The use of neodymium-doped yttrium aluminum garnet (Nd:YAG) laser as a treatment for CL was reported in one study in which the authors compared Nd:YAG laser therapy with intralesional MA^25^. They treated the CL lesions of 16 patients simultaneously, treating one lesion with 1,500 mg/5 μl of MA weekly (until complete recovery of the lesion) and another lesion with Nd-YAG laser (200 mJ/cm^2^, pulse duration 20 ms, spot size 3 mm indexes). The sessions with Nd-YAG laser were repeated at intervals of 2 weeks until complete cure with a negative direct smear of the lesion. The mean number of sessions with Nd-YAG was 2.56 ± 0.89 and for MA injection was 7.31 ± 4.01 (*P* <0.001). Scars were observed in 10 patients of the Glucantime-based treatment group and only three patients of the laser group. The laser scars were smaller but were more often associated with post-inflammatory hyperpigmentation. The authors concluded that laser therapy might be an effective alternative treatment for CL with fewer complications^25^.

***Pulsed dye laser*.** The mechanism of action of pulse dye laser (PDL) on CL is not completely understood; however, it is believed that cutaneous lesions have an important vascular component owing to the identification of dermoscopic vascular patterns in 100% of leishmania lesions reported in a study, a fact that could explain the efficacy of PDL. Other possible mechanisms of action are the heat ablation of the *Leishmania* bodies, following the basis of thermotherapy, and the cutaneous immunological activation induced by the PDL^26^. Slaoui *et al*. used PDL 595 nm to treat the erythematous papules and nodules of leishmania in three patients using the following parameters: spot size 10 mm and energy 8 J/cm^2^. They reported improvement in texture and scar size^26^. Elsaie and Ibrahim treated 25 cutaneous lesions of 12 patients with a single pass over the whole lesion using a fluence of 7 J/cm^2^, 10 mm spot size, and a pulse duration of 0.45 ms; they reported full cure in 13 of the 25 lesions after three sessions, while 12 of the remaining 25 lesions required four sessions toward complete recovery. They reported that PDL is an effective and safe treatment^27^.

### Topical paromomycin

Paromomycin (formerly aminosidine) is an antibacterial aminoglycoside available in parenteral and 15% topical formulations that has efficacy against *Leishmania*^28,29^. It has been used in topical formulations; one of these is the combination of 15% paromomycin with 12% methylbenzethonium chloride, which, when applied for 20 days, showed a 77% efficacy compared to 27% for placebo, but severe irritancy and intolerance has been associated with methylbenzethonium^29,30^. A study conducted in Panama similar to a trial conducted in Tunisia used a new formulation (paromomycin-gentamicin cream), which demonstrated a cure rate of 79% against New World *Leishmania* species, with mild local adverse events^29,31^. It is important to emphasize that the results from the Tunisia and Panama studies were obtained using a different formulation than the one used in previous studies, where either methylbenzethonium chloride or urea was used as a vehicle and use had to stop owing to side effects. This problem about safety has not been observed with the new formulation used in Panama and Tunisia.

## Systemic therapy

### Antimonials

**Figure 1.**
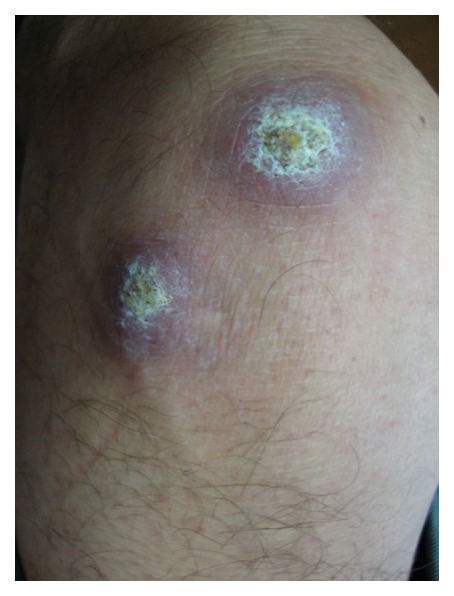
Cutaneous leishmaniasis in a Mexican patient.

**Figure 2.**
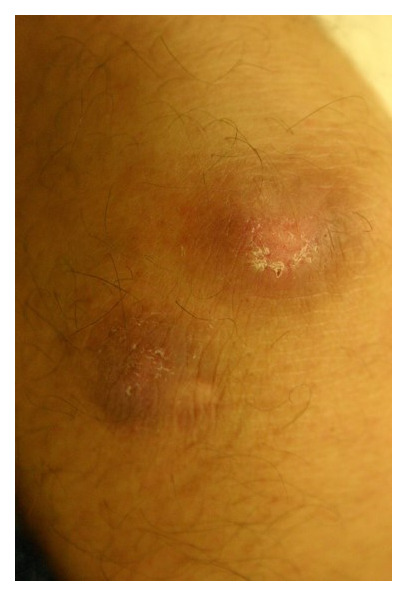
After four doses of intralesional meglumine antimoniate.

***Meglumine antimoniate and sodium stibogluconate*.** SbV compounds are highly effective and the first-line treatment for most forms of leishmaniasis; these are SSG (Pentostam®) and MA (Glucantime®)^18,28^. MA is administered intramuscularly or intralesionally (Figure 1 and Figure 2) and SSG is administered intravenously or intralesionally at the recommended dose of 20 mg/kg/day for 20 days^28^. They have many adverse effects, the most frequent of which are cardiotoxicity, rise in liver function test, urea, and creatinine, anorexia, nausea, vomiting, myalgia, and arthralgia, whereas when applied intralesionally they may cause local irritation, pain, edema, erythema, or pruritus^1,8^. Both intralesional and systemic antimonials are recommended by the WHO and PAHO for CL treatment^32,33^. Castelano *et al*. in a systematic review reported similar efficacy rates of intralesional SbV use in Old and New World CL. They also found that treatments with a duration of more than 14 days had higher cure rates^7^. A systemic antimonial is indicated for mucocutaneous forms, with a cure rate of almost 75%, which depends on the causal species and the severity of the disease. The recommended dosage is 20 mg/kg/day for 28–30 days^1,8,28^.

### Antifungals

The azole molecules (imidazoles and triazoles) block the ergosterol synthesis of *Leishmania* parasites. Ketoconazole, itraconazole, and fluconazole have been used for CL in many studies with variable cure rates^8,34^. Important advantages of azoles are oral administration and fewer side effects^28^. A systematic review and meta-analysis which included studies involving outcomes showed a pooled azole final efficacy rate of 64% for the treatment of CL, finding a higher healing rate in *Leishmania mexicana* (89%), *Leishmania infantum* (88%), and *Leishmania donovani* (80%) and lower cure rates for *Leishmania major* (53%), *Leishmania braziliensis* (49%), and *Leishmania tropica* (15%)^35^. A randomized controlled trial in India compared oral itraconazole 200 mg/day for 6 weeks versus no treatment and showed complete cure in 66.7% of the itraconazole group and none of the placebo group^18^. In Italy, pediatric patients with OWCL were treated with fluconazole at a dose of 6 mg/kg daily for 6 weeks and exhibited complete cure^36^. Long-term treatment with azoles have toxicity risks, such as hepatotoxicity and QTc prolongations, which is why the recommended dosage of fluconazole is 200 mg or 400 mg daily for 6 weeks, ketoconazole 600 mg daily for 28 days, and itraconazole 100 mg twice daily for 42–56 days^3^.

***Amphotericin B*.** Amphotericin B (AmB) is a macrolide polyene antibiotic with potent antifungal and anti-leishmanial activity that targets ergosterol, the principal membrane component of *Leishmania* spp.^37,38^. It is considered a second-line treatment for CL. There are four formulations of AmB: one is amphotericin B deoxycholate, and the rest are lipid formulations of the drug, liposomal amphotericin, cholesterol dispersion amphotericin, and lipid complex amphotericin^2,8,37^. The three lipid formulations were made to minimize adverse events such as nephrotoxicity reported with amphotericin B deoxycholate; however, the only drug of this type approved by the US Food and Drug Administration (FDA) is liposomal amphotericin B (L-AmB)^8^. L-AmB has an efficacy of 95–100% in visceral leishmaniasis^39^. However, there are a few studies of CL treatment with L-AmB, and they show big differences in the reported cure rates and the total doses used^8^. A review of L-AmB treatment of OWCL reported that L-AmB had an 85% cure rate in immunocompetent patients with OWCL but also found a high variety of total treatment dosages, which limits the reported data, so the authors concluded that there is a need for randomized controlled trials comparing systemic treatments^39^.

***Miltefosine*.** Miltefosine is an alkyl phospholipid and is the only oral drug for the treatment of leishmaniasis that is approved by the FDA. It was approved in 2014 as a treatment for CL caused by *L. braziliensis, Leishmania panamensis,* and *Leishmania guyanensis*. The efficacy results from clinical trials, most of them for New World CL, have high variability between countries and the different *Leishmania* species. The reported cure rates range from 50% *(L. braziliensis* in Colombia*)* to 88% *(L. major* in Afghanistan)^8,40,41^. The FDA regimen is 2.5 mg/kg/day for 28 days, with a maximum dose of 150 mg/day owing to poor gastrointestinal tolerability. The main disadvantage of miltefosine treatment is the high incidence of gastrointestinal adverse events, the most common of which are vomiting and diarrhea (62% of patients). Hepatic toxicity and nephrotoxicity can occur in 10–15% of patients. It is also teratogenic; thus, it requires women of childbearing age to avoid pregnancy during treatment and for 3 months after finishing treatment. Moreover, it has a long half-life, which makes it vulnerable to the development of drug resistance^8,40–42^.

***Pentamidine isethionate*.** Pentamidine is an antiprotozoal and antifungal agent of the group of aromatic diamidines that can be administered intravenously or intramuscularly in leishmaniasis^18^. It is exclusively used for *L. guyanensis*. The recommended dose is 4 mg/kg/day on alternate days (maximum dose of 240 mg/day)^3,8^. Christen *et al*. reported a higher efficacy of the intravenous administration of pentamidine isethionate (85.3%) versus intramuscular administration (51.3%) in *L. guyanensis* CL^43^. However, it is generally used as a second-line treatment owing to nephrotoxicity, hepatotoxicity, pancreatitis leading to insulin-dependent diabetes, hypertension, hypoglycemia, QT prolongation, hyperkalemia, and vertigo^1,3,8^.

### Antidepressants

***Sertraline*.** Sertraline is an interesting example of repurposing drugs for treatment. It is a selective serotonin reuptake inhibitor (SRRI) prescribed for depression and anxiety. Lima *et al*. assessed the detrimental effects of sertraline on *L. infantum* and how it inhibited the proliferation of promastigotes with IC_50_ and IC_90_ values of 2.0 ± 0.7 µM and 8.4 ± 1.8 µM, respectively, and intracellular amastigotes with IC_50_ and IC_90_ values of 3.9 ± 0.3 µM and 7.9 ± 0.1 µM, respectively. Sertraline had no toxic effects on murine macrophages^44^.

Romanella *et al*. assessed the efficacy of sertraline entrapped in phosphatidylserine liposomes (LP-SERT) in a murine model of visceral leishmaniasis. This treatment was able to reduce the liver parasite burden by 72% at 0.3 mg/kg (*P* <0.05) and by 89% at 1 mg/kg (*P* <0.05) when compared with the placebo group (only liposomes), who experienced no effect^45^.

Clomipramine is a tricyclic antidepressant used for some psychiatric disorders. Da Silva Rodrigues *et al*. assessed its activity in *Leishmania amazonensis* and found that it was a selective inhibitor of the intracellular and extracellular forms. It inhibited macrophage cellular growth by 50%. It appears to induce morphological alterations, oxidative stress, mitochondrial impairment, and autophagy, so further studies are needed in animal models^46^.

### Amiodarone

Amiodarone is a potent antiarrhythmic whose anti-leishmanial activity was reported recently in different studies^47,48^. The mechanisms of action as an anti-leishmanial are inhibition of sterol biosynthesis, destabilization of Ca^2+^ homeostasis, the collapse of mitochondrial membrane potential, and production of reactive oxygen species^49,50^. In a study from Iran, researchers studied the effectiveness of amiodarone against *L. major* and reported that it reduced the lesion surface area but didn't result in complete cure, suggesting that combination therapy with amiodarone may yield better results^49^.

### Immunomodulators

***Cyclosporin A and dihydrocyclosporin A*.** Cyclosporin A (CsA) exhibits its immunosuppressive action by inhibiting the production of calcineurin through binding to cyclophilin A (CyPA). *L. donovani* expresses a variant of CyPA (LdCyPA) that is different to those of humans and has a role in the survival of parasites in the tissues^51^. Dihydrocyclosporin A (DHCsA-d) (a co-metabolite of CsA) has minimum immunosuppressive activity and also inhibits *L. donovani in vivo* and *in vitro*. Zheng *et al*. evaluated the efficacy of both drugs against promastigotes and amastigotes of *L. donovani* versus SSG as a positive control. Intracellular amastigotes decreased compared to the untreated group after the administration of DHCsA-d or SSG^51^. Also, the intracellular amastigotes increased after the administration of CsA, and it appears that only the last was found to promote intracellular amastigotes^51^.

***Imiquimod*.** Imiquimod can be used in combination with standard therapy with cure rates above 90%. It appears to interact with the Toll-like receptors and induces activation of the NF-κB pathway, thus inhibiting amastigote replication. It also induces the production of nitric oxide (NO) by macrophages, which could lead to *Leishmania* destruction^52^.

Verastegui-Miranda *et al*. led a randomized double-blind trial comparing treatment outcome with pentamidine and the outcome with pentamidine intralesional infiltration + imiquimod (three times/week for 20 days). They found a cure rate of 75% in the pentamidine + imiquimod group versus 58% in the pentamidine alone group^53^. The same group also led a randomized double-blind study in 2005 for treatment with imiquimod versus Glucantime. They found that the group treated with imiquimod reached cure faster (50% achieved cure at 1 month versus 15% in the placebo group). As for the adverse events, erythema was more frequent in the imiquimod group^54^.

Arevalo *et al*. used imiquimod as monotherapy for *Leishmania peruviana* and showed a transient effect with minimal reduction of the lesion size in the first few days but without cure^55^. Mejravaran *et al*. studied the effects of imiquimod liposomes containing *Leishmania* antigens in a murine model. The smallest lesions at the end of the study were observed in mice who had received liposomes + imiquimod compared to other mice (*P* <0.05)^56^.

## Alternative treatments

### Plant-derived treatments

Chalcones (benzylideneacetophenones or 1,3-diaryl-2-propen-1-ones) are plant metabolites and precursors of isoflavonoids and flavonoids. At least 312 compounds with the chalcone skeleton have anti-leishmanial activity, 34 derived from plants of the Fabaceae and Piperaceae. All of them, natural and synthetic or semisynthetic, showed varying degrees of this activity^57^. An extract of *Piper aduncum* (Piperaceae) led to the isolation of 2',6'-dihydroxy-4'-methoxychalcone (DMC2), which inhibited promastigotes of *L. amazonensis* but had less of an effect on amastigotes. There were no toxic effects on macrophages^57^.

***Arrabidaea brachypoda*.** Three flavonoids from *A. brachypoda* have shown anti-*Trypanosoma cruzi* activity. Rocha *et al*. investigated the anti-leishmanial activity of three dimeric flavonoids *in vitro* and selected the most active (Brachydyn2) for *in vivo* testing in a model of CL in which treated amastigotes showed cell lesion induced by Brachydyn 2, which led to death of the parasite^58^.

***Styrylpyrone*.** Extracts from species of the Lauraceae family of the Amazon have shown *in vitro* anti-leishmanial activity. Styrylpyrone 4-methoxy-6-(11,12-methylenedioxy-trans-styryl)-2-pyrone (SP) derived from *Aniba panurensis* was evaluated *in vitro* against *L. amazonensis* promastigotes, which were treated with different concentrations of SP, affecting the growth of the parasite^59^. The mechanism of action involves impairment in cell division and, in an attempt to maintain homeostasis, the parasite will initiate an autophagic process, leading to cell collapse and death^59^.

***Piper marginatum*.** Essential oils from *Piper marginatum* showed phenolic compounds, terpenoids, and 3,4-methylenedioxypropiophenone as major compounds. These fractions have antipromastigote and antiamastigote properties against *L. amazonensis in vitro*. They showed low toxicity for macrophages and had the best selectivity index versus pentamidine (reference drug)^60^.

***Origanum onites*.** The essential oil of *Origanum* species has shown antimicrobial properties, a few of them evaluated as antiprotozoal. Tasdemir *et al*. assessed the antiprotozoal activity of *Origanum onites* against some protozoa^61^. The main components of the oil were carvacrol (70.6%), linalool (9.7%), *p*-cymene (7%), *γ*-terpinene (2.1%), and thymol (1.8%). It had *in vitro* activity against *Trypanosoma brucei rhodesiense* and moderate anti-leishmanial and anti-plasmodial effects. It did not show toxicity to mammalian cells. Some of these compounds were assessed individually with a decrease in their activity, suggesting that they have an additive action^61^.

***Urtica dioica*.** Badirzadeh *et al*. studied the anti-parasitic effects of *Urtica dioica* aqueous extract in CL caused by *L. major in vivo* (mouse model) and *in vitro*. The results demonstrated that the optimal concentration for reducing amastigote and promastigote growth was 3,500 and 6,000 μg/mL, respectively, killing both forms of the parasite. This study proves that *U. dioica* has potent action against *Leishmania* promastigotes and amastigotes. The possible mechanism of action is via modulation of the immune response. It did not show toxicity in macrophages^62^.

### Animal toxin-derived treatments

***Snake-derived venoms*.** Snake venoms are a complex mixture of peptides which have different pharmacological activities^63^. Venoms from *Bothrops moojeni* can inhibit the *in vitro* growth of *Leishmania* spp. because of L-amino acid oxidase^63^. *Crotalus durissus terrificus* (South American rattlesnake) has a variety of peptides in its venom: gyroxin, crotamine, convulxin, crotoxin, and others^63^. Crotoxin is the major toxic compound of this venom, and it has phospholipase A2 (PA2). PA2 from other snakes has action against promastigotes of *Leishmania* spp.^63^. Barros *et al*. showed that the PA2 isolated from *C. d. terrificus* inhibits the proliferation of *L. infantum chagasi* promastigotes at 50–200 μg/mL and that it was dose dependent. It appears that the interaction between PA2 and the lipid bilayer causes a rupture in the membrane, and this acts as an inhibitor of promastigote proliferation. It also induces the production of NO by macrophages (some *Leishmania* species can inhibit its production, escaping this mechanism of destruction), which can help the macrophages to suppress the infection in early stages^63^.

Macedo *et al*. encapsulated the toxin of *C. d. terrificus* in a polymeric microparticle system and tested its activity in mice. They found that macrophages could better internalize the microparticle system versus toxin alone but both of them could promote the production of TNF-α, which has an important intracellular role in inducing the production of NO and peroxide ions. The group with the microparticle system was able to decrease the number of amastigotes (50.8%) versus the group without the toxin (14.9%)^64^.

## Vaccines and immunotherapy

It is possible to recover from primary infection caused by *L. major* and *L. braziliensis*, and the latter is associated with long-term protection against reinfection, indicating that a vaccine can be developed against CL. The interaction between the parasite and dendritic cells is crucial to develop CD4 and CD8 lymphocyte activation. This interaction leads to a release of IFN-γ that induces the production of NO by macrophages. The vaccine is formed by two compounds: the antigen, to generate a specific immune response, and an adjuvant, which initiates and directs the immune response.

Five first-generation vaccines have been approved against *Leishmania*, two of them for administration to humans, and these vaccines use the whole parasite in either live or attenuated form (Brazil used it as immunotherapy for CL using killed *L. amazonensis* parasites and Uzbekistan used live attenuated parasites of *L. major*). There is also the risk of inducing the disease while using whole living parasites or introducing new species in non-endemic areas^65^.

Three vaccines of second generation (using recombinant antigens and specified adjuvants) have been developed for the immunization of dogs (Brazil). These three vaccines have proven to be effective in the short term, but the long-term efficacy remains unknown, so further studies are needed to know if it can reduce the spread of this disease. If used for immunotherapy, they need to be used in combination with traditional treatment. It would also need to be given in multiple injections to induce a satisfactory immune response^65^.

GP63 metalloproteinase is a protein on the surface of *Leishmania* that mediates protection against *L. mexicana* and *L. major* on mice but only partial protection in monkeys^65^.

Third-generation vaccines use specific pathogen RNA or DNA or a carrier that contains some gene components of the parasite to induce an immune response; it has been shown that the ones that use DNA are better at inducing the immune response than those using RNA, but they only showed acceptable results in mice because it is needed at a higher dose of DNA in larger animals and humans to induce the same immune response^65^.

LACK is the most-studied DNA vaccine against *Leishmania*; its use combined with IL-12 increased protection compared with LACK alone^65^.

There are no approved vaccines for human CL, but those that have advanced to some clinical trials (two vaccines, killed *L. amazonensis*, first generation) had inconsistent results between placebo and vaccine groups, so further studies are needed. There is a polyprotein-containing subunit vaccine (LEISH-F1+MPL-SE) that appears to be safe, and it generates an antigen-specific Th1 lymphocyte response^65^. The most-evolved vaccines are based on recombinant molecules (LEISHF1, LEISHF2, and LEISHF3 have already entered human clinical trials, second-generation vaccines). LEISHF1 could efficiently treat CL and mucosal leishmaniasis, but it can also induce immunity in healthy individuals. LEISHF2 entered phase II trials, which are assessing its safety and therapeutic effect on CL patients versus chemotherapy alone^66^. The existence of veterinary vaccines and the development of clinical trials related to human disease are an indication that it is possible to develop a human vaccine. However, it is a difficult task, and this relies on the interaction between the host and parasite, which is complex^66^.

Immunotherapy had success in patients with mucocutaneous and diffuse CL. Moafi *et al*. treated promastigotes of *L. braziliensis* killed by pasteurization associated with viable Bacillus Calmette–Guérin (BCG), offering a safe option in disseminated CL which did not respond to conventional treatment^67^.

There is a third-generation vaccine employing adenovirus (ChAd63) that can induce a CD8^+^ response against *Leishmania* antigens and promotes the secretion of IFN-γ by dendritic cells, which can lead to the prevention and treatment of *Leishmania* infection; it is still in phase II of a non-controlled randomized trial. It was focused as a single-dose therapeutic agent, and a single dose was safe and induced an adequate CD8^+^ response^68^.

## Conclusions

Leishmaniasis remains a major public health problem with a broad spectrum of clinical manifestations related to the immune response of the host. New drugs are proposed for treatment, but results are still unsatisfactory. Currently, pentavalent antimonials are effective and available, but research must be a cornerstone in the proposal of novel therapies.
